# Supplementary material for: Crystallization Mechanism and Optical Properties of Antimony-Germanate-Silicate Glass-Ceramic Doped with Europium Ions
Source: Materials (Basel). 2022 May 26;15(11):3797. doi: 10.3390/ma15113797 (PMC9181671; doi:10.3390/ma15113797)
Supplement: Supplementary file 1 [file materials-15-03797-s001.zip › materials-1734586-supplementary.pdf]

Supplementary Materials:

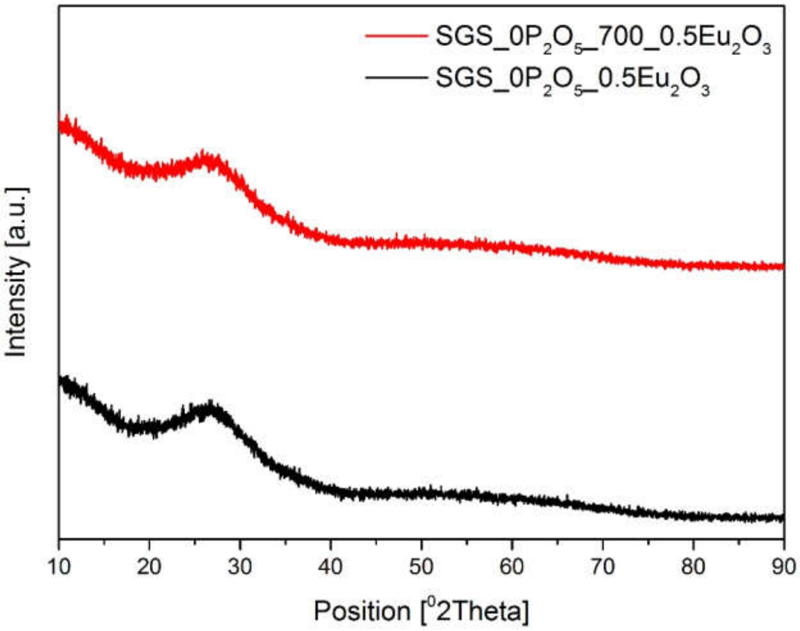

Figure S1. XRD patterns of SGS (without P_2O_5).

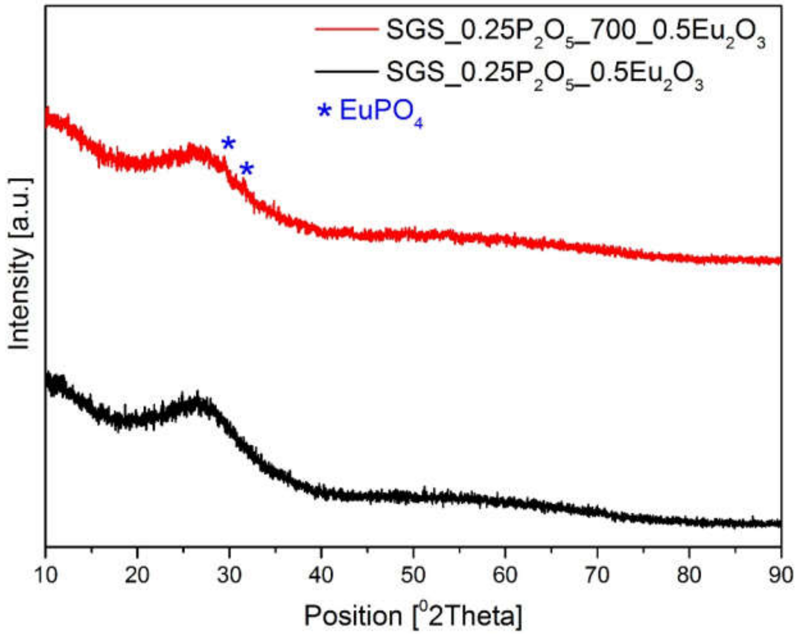

Figure S2. XRD patterns of SGS (with 0.25% P_2O_5).

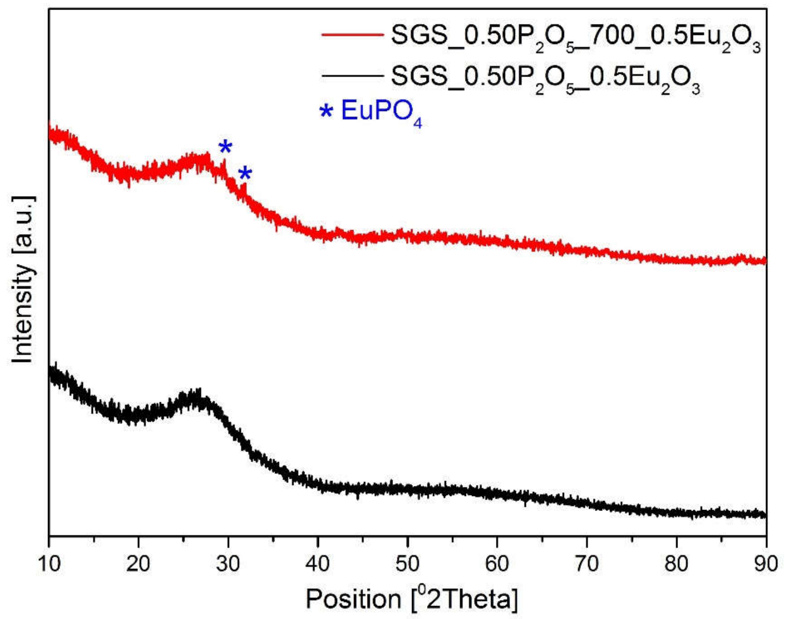

Figure S3. XRD patterns of SGS (with 0.5% P_2O_5 dope).

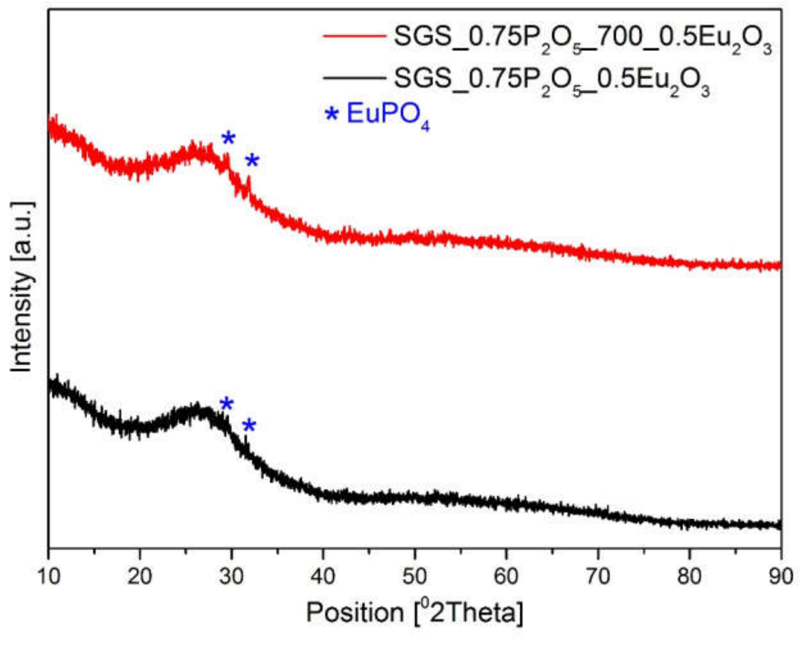

Figure S4. XRD patterns of SGS (with 0.75% P_2O_5 dope).

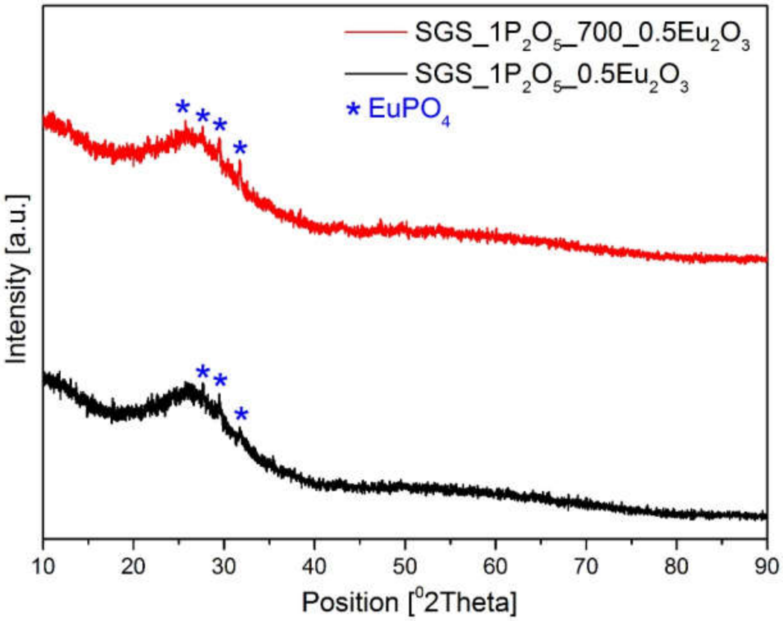

Figure S5. XRD patterns of SGS (with 1.0% P_2O_5 dope).

The size of the crystallites was determined from the Scherrer formula:

$$D_{hkl} = \frac{k\lambda}{\beta \cos \theta},$$

where:

D_{hkl} - average crystallite size [nm]];

k - Scherrer's constant;

λ - X-ray wavelength, = 0.15406 [nm];

β - half-width of the reflection [rad].
